# Prevalence of diabetes and predictions of its risks using anthropometric measures in southwest rural areas of China

**DOI:** 10.1186/1471-2458-12-821

**Published:** 2012-09-24

**Authors:** Xiaolong Zhao, Xiaoming Zhu, Hengsheng Zhang, Weiwei Zhao, Jinhui Li, Yonghui Shu, Songwu Li, Minghui Yang, Linghu Cai, Jiping Zhou, Yiming Li

**Affiliations:** 1 Department of Endocrinology & Metabolism, Huashan Hospital, Medical College Fudan University, Shanghai, China; 2 Department of Internal Medicine, People’s Hospital, Songming County, Yunnan Province, China; 3 Center for Disease Control and Prevention of Songming Country, Yunnan Province, China

**Keywords:** Obesity, Waist-to-height ratio, Body mass index, Waist hip ratio, Diabetes type 2

## Abstract

**Background:**

To examine the prevalence of diabetes and prediabetes in Songming county, Yunnan province, South-west China and examine influences of anthropometric indicators on diabetic risk.

**Methods:**

This study was a population based cross-sectional study of 1031 subjects in Songming County aged 30 years and older. Age-standardization was performed by using the 2010 Songming population as the standard population. After an overnight fasting, participants underwent an oral glucose tolerance test (OGTT), and venous blood glucose levels were measured to identify diabetes and prediabetes. Physicians completed questionnaires and blood pressure measurements; trained nurses measured anthropometric variables. Age-adjusted logistic regression models were used to assess the association between anthropometric variables and diabetes.

**Results:**

Total prevalences of diabetes and prediabetes were 10.0% and 11.6%, respectively. In women, prevalence of diabetes and prediabetes significantly increased with body mass index (BMI),waist hip ratio (WHR), and waist-to-height ratio (WHtR). But in men, prevalence of diabetes and prediabetes only significantly increased with WHR and WHtR. Compared to 1^st^ WHR tertile in women, there was a nearly tenfold increase in the risk of diabetes with 3^rd^ WHR tertile (OR 10.50, 95% CI 3.95-27.86). Men with 3^rd^ BMI tertile had 4.8-fold risk of getting diabetes compared to men with 1^st^ WHtR tertile (OR 4.79, 95% CI 1.88-12.26). Only WHtR had significantly higher receiver operating characteristic (ROC) area than BMI in total men (0.668 vs. 0.561, p < 0.05). And in total women, only WHR had significantly higher ROC area than BMI (0.723 vs. 0.628, p < 0.05). In the partial correlation analysis controlling for waist circumference, only WHR had significant correlation with fasting plasma glucose (r = 0.132, p = 0.002) and 2-h plasma glucose (r = 0.162, p = 0.000) in women, and WHtR had a much stronger association with both fasting plasma glucose (r = 0.305, P = 0.000) and 2 h plasma glucose (r = 0.303, P = 0.000) than WHR in men.

**Conclusion:**

High prevalence of diabetes and prediabetes were found in this underdeveloped region. About half of total subjects with diabetes were undiagnosed. The association of obesity indices and diabetic risk factors varied with gender. The strongest predictors of diabetes were WHR for the female subgroup and WHtR for the male subgroup.

## Background

Diabetes is a major public health problem in the world. The total number of diabetic patients is expected to soar to 366 million in 2030 [1]. The disease has caused a huge social and economic burden. Detailed information about the prevalence and demographics of diabetes in specific area was important for health care providers to take suitable approaches [2]. In a recent study, Yang et al. [3] estimated that 92.4 million adults 20 years of age or older (9.7% of the adult population) had diabetes, which was 4.5 times the number estimated in the Chinese National Nutrition and Health Survey in 2002 [4]. But the study by Yang et al. has aroused widespread controversy. Rural residents were undersampled and no participants were recruited from southwesten area of China, such as Yunnan province [3].

Yunnan is adjacent to Southeast Asia Region (SEAR) bordering Myanmar, Laos and Vietnam. Yunnan province shares some typical characteristics of SEAR, such as ethnic feature, cultural feature and agricultural economy [5]. Yunnan is one of China’s poorest provinces and is located in a mountainous area at an average elevation of 2000 ~ 3000 m. In 2000, 87% of the population were peasants and the illiteracy rate was 50%. It boasts the biggest-selling Chinese cigarette brands and has the largest tobacco production in China [6]. The local inhabitants have a high prevalence of tobacco smoking and like salty and greasy food.

In Yunnan, the economy has traditionally been dominated by agriculture and more than 60% of the population live in rural areas. It has been considered the typical representation of underdeveloped regions in China. On the other hand, on the background of China’s western region development strategy in recent years, lifestyle and social structure began to change dramatically. Therefore,understanding the epidemic characteristics of diabetes is important for healthcare providers to formulate appropriate public health policies in this rural area. So far, however, no data has been reported on the prevalence of diabetes in this region.

This study was designed to provide reliable data on the prevalence of diabetes and prediabetes in Songming County and to identify the best anthropometric indices in predicting risk of diabetes.

## Methods

### Study design

This was a population-based, cross-sectional study in Songming County from June 2010 to September 2010. Information on personal characteristics, anthropometric indices, blood glucose levels and blood pressures was collected.

### Study area and population

Our study was conducted in Songming County, Yunnan province, southwest China. It is in the middle of Yunnan province (longitude 103.03E, latitude 25.35 N). Per capita disposable income in the county was 2230 yuan in 2001 and 5162 yuan in 2010. (In 2010, 6.7695 yuan equaled U.S. $1.00.). In 2010, it had a population of 292250 and contained 8 townships. The total area is 1357.29 km^2^. Villages are scattered with average distance of 43 km from Kunming, capital of Yunnan Province.

### Sampling technique

A multistage stratified sampling method was used to select a representative sample of persons 30 years of age or older. Firstly, all townships were divided into three groups including county, industrial town and agricultural town according to their geographic regions and economic development status. Secondly, three villages were randomly selected from each group. In each selected village, individuals aged ≥30 years were recruited, all whom were Han Chinese and had homogeneous lifestyles. All the subjects had lived in Songming County for 5 years or longer [3,7].

### Data collection

The survey was conducted in a given site of each village. Physicians completed questionnaires and blood pressure measurements, and trained nurses measured anthropometric variables including height, weight, waist circumference and hip circumference and performed OGTT. The questionnaires included questions related to the diagnosis and treatment of diabetes. If participants had a history of diabetes or were taking drugs to treat diabetes, steamed bread meal tests were performed. If not, an OGTT was performed to diagnose diabetes. Participants who were suspected of undiagnosed diabetes were told to go to Songming County People’s Hospital to undergo another OGTT to confirm the diagnosis. No individual was found to take metformin for other reasons than diagnosed diabetes.

### Oral glucose tolerance test

After an overnight fasting for at least 10 h, a standard 75-g glucose solution was given. And venous blood samples were drawn at 0 min and 120 min (2-h) to identify diabetes and pre-diabetes (impaired glucose tolerance and/or impaired fasting glucose). Venous blood samples collected from the antecubital vein were put into vacuum tubes containing sodium fluoride in 4°C ice boxes and were analyzed within 3 h. Samples of venous plasma glucose were measured with the use of an oxidase enzymatic method on a laboratory automatic analyzer (Hitachi 7080, Tokyo, Japan).

### Steamed bread test

According to Chinese medical ethics, for participants with previously diagnosed diabetes, steamed bread tests were preformed [3]. In this test, a 100-g steamed bread contained approximately 75-g of complex carbohydrates were given to test glucose tolerance or β-cell function [8]. Blood samples were drawn at 0 min and 120 min after the carbohydrate load. Venous blood glucose after this load has been shown to be linearly related to glucose measured 120 min after an OGTT [9].

### Blood pressure measurements

Physicians performed blood pressure measurements using an American Heart Association protocol [10]. After 5 min of rest in a sitting position, systolic and diastolic pressures were measured from the participant’s right arm using standard mercury sphygmomanometer. Two successive measurements were performed with at least one-minute interval in between.

### Anthropometric measurements

Anthropometric measurements included height, weight, waist circumference and hip circumference. Body mass index (BMI) was calculated as weight (kg) divided by height squared (m^2^). WHtR was calculated as height (cm) divided by waist circumference (cm). WHR was calculated as hip circumference (cm) divided by waist circumference (cm). Each of these measurements was completed by two nurses; one took the measurements, the other recording the readings. The height and body weight were measured simultaneously with the participants standing on a balance beam scale with the participants wearing light clothing and no shoes. Hip circumference was measured at the level of maximal gluteal protrusion [11]. Waist circumference was measured with subjects standing relaxed and in underclothes only. Waist circumference was obtained at the midpoint between the anterior superior iliac crest and the lowest rib. The upper border of the iliac crest and lowest rib were located, and a plastic anthropometric tape was wrapped around above this point, to ensure it was adjusted without compressing the skin. And the reading was taken at the end of a normal breath [11].

### Diagnostic criteria

Total diabetes included both diagnosed and undiagnosed diabetes (Table 1). The diagnosis of undiagnosed diabetes and prediabetes was based on the 1999 WHO diagnostic criteria [12]. Prediabetes was defined as either impaired fasting glucose (IFG) or impaired glucose tolerance (IGT).

**Table 1:** Diagnostic criteria for total subjects

**Subjects**	**Test**	**Category**	**Diagnostic criteria**	**Venous plasma glucose ** (**mmol/L**)
History of diabetes	Steamed bread test	Diagnosed diabetes ^a^	A positive response to the question, “Has a doctor ever told you that you have diabetes?”	
No history of diabetes	OGTT [10]	Undiagnosed diabetes	Fasting	≥7.0
			2-h	≥11.1
		Isolated IGT	Fasting	<6.1
			2-h	≥7.8 and <11.1
		Isolated IFG	Fasting	≥6.1 and <7.0
			2-h	<7.8
		Combined IFG and IGT	Fasting	≥6.1 and <7.0
			2-h	≥7.8 and <11.1
		Normal	Fasting	<6.1
			2-h	<7.8

*IGT*, impaired glucose tolerance.

*IFG*, impaired fasting glucose.

^a^ The diagnosed diabetes was identified by a positive response to the following question, “Has a doctor ever told you that you have diabetes?” [3].

### Statistical analysis

The population was stratified by age into six groups. Anthropometric indices including BMI, WHR and WHtR were equally divided into three groups (1^st^, 2^nd^ and 3^rd^ tertile). The continuous variables were expressed as mean ± standard deviation. Chi-square tests were used for categorical variables, and Student’s t tests were used to assess differences in continuous variables. Age- and gender-specific crude prevalence were standardized to the overall age distribution of Songming population in 2010. Logistic regression was used to determine the impact of anthropometric indices on diabetes. In an epidemiological study, this attributable risk provides an estimation of public health impact of these anthropometric indicators [13]. P < 0.05 was considered to be statistically significant and all P values were two-sided. ROC curves were used to determine the predictive power of each anthropometric indices. All statistical analyses were performed using the Statistics Package for the Social Sciences software release 15.0 (SPSS, Chicago, IL, USA).

### Ethical approval

Written informed consents were obtained from all participants and the study was approved by the Institutional Review Board of Songming County People’s Hospital.

## Results

### Sample characteristics

Basic characteristics were summarized in Table 2. 1031 subjects were recruited in this study, and there were more females (52.7%) than males (47.3%). The mean age was higher in men (55.4 years) than women (54.0 years). A total of 120 diabetic patients were enrolled, of whom 63 (52.5%) were diagnosed and 57 (47.5%) undiagnosed. Among the diabetic subjects, 59.2% were women and 40.8% were men.

**Table 2:** Characteristics of study participants

**Participants**	**Normal (n=768)**	**Prediabetes (n=143)**	**Undiagnosis diabetes(n=57)**	**Diagnosis diabetes(n=63)**	P^*^
Gender, n (%)					0.039
Female	394(62.6%)	78(14.4%)	28(5.2%)	43(7.9%)	
Male	374(76.6%)	65(13.3%)	29(5.9%)	20(4.1%)	
Age, n(%)					0.000
<50	327(83.6%)	34(8.7%)	15(3.8%)	15(3.8%)	
50-65	319(72.3%)	64(14.5%)	24(5.4%)	34(7.7%)	
>65	122(61.3%)	45(22.6%)	18(9.0%)	14(7.0%)	
BMI (kg/m2) ^a^					0.000
<25	529(79.9%)	71(10.7%)	24(3.6%)	38(5.7%)	
≥25	239(64.8%)	72(19.5%)	33(8.9%)	25(6.8%)	
Waist circumference ^b^					
Male <90 cm	307(79.1%)	44(11.3%)	21(5.4%)	16(4.1%)	0.096
Male ≥ 90 cm	67(67%)	21(21%)	8(8%)	4(4%)	
Female <80 cm	272(81.9%)	33(9.9%)	7(2.1%)	20(6.0%)	0.000
Female ≥80 cm	122(57.8%)	45(21.3%)	21(10.0%)	23(10.9%)	
WHR ^b^					
Male < 0.9	271(82.1%)	33(10.0%)	15(4.5%)	11(3.3%)	0.001
Male ≥ 0.9	103(65.2%)	32(20.3%)	14(8.9%)	9(5.7%)	
Female F <0.85	212(88.0%)	16(6.6%)	6(2.5%)	7(2.9%)	0.000
Female ≥0.85	182(60.3%)	62(20.5%)	22(7.3%)	36(11.9%)	
WHtR ^c^					
< 0.5	460(82.6%)	52(9.3%)	18(3.2%)	27(4.8%)	0.000
≥ 0.5	308(65.0%)	91(19.2%)	39(8.2%)	36(7.6%)	
SBP					
<140 mm Hg	607(78.6%)	87(11.3%)	34(4.4%)	44(5.7%)	0.000
≥140 mm Hg	607(78.6%)	87(11.3%)	34(4.4%)	44(5.7%)	
DBP					
<90 mm Hg	661(76.7%)	107(12.4%)	43(5.0%)	51(5.9%)	0.008
≥90 mm Hg	107(63.3%)	36(21.3%)	14(8.3%)	12(7.1%)	

Data are expressed as n(%).

* Chi-square tests for trend.

^a^ According to the cut-offs as suggested by WHO [29].

^b^ According to the cut-offs as suggested by WHO [41].

^c^ According to the cut-offs as suggested by He et al. [42].

*WHR*, waist hip ratio.

*WHtR*, Waist-to-height ratio.

*BMI*, body mass index.

*SBP*, systolic blood pressure.

*DBP*, diastolic blood pressure.

### Age- and gender- specific prevalence of diabetes and prediabetes

The crude and age-standardized prevalences of diabetes and prediabetes among men and women were shown in Table 3, respectively. The prevalences of diabetes and prediabetes rose with age in women. Age-specific prevalence of diabetes in men increased progressively with increasing age, but fell in 60–69 age group. Pprevalence of prediabetes in men also fell in 50–59 age group (Table 3).

**Table 3:** Age and gender prevalence of different intolerance categories among Techranian adults

**Age group (years)**	**Study population (n)**	**Isolated IFG (%)**	**Isolated IGT (%)**	**IFG/IGT (%)**	**Undianosed diabetes (%)**	**Known diabetes**
**Men**						
20-29	694	5.1	1.0	0.7	0.8	1.1
30-39	1054	9.8	4.4	1.7	1.6	2.8
40-49	775	11.1	6.9	4.6	5.2	6.9
50-59	605	11.2	7.0	7.3	9.3	14.7
60-69	637	8.5	8.2	9.4	8.6	20.3
≥70	241	7.1	11.7	6.2	14.9	19.1
Un-standardized	4006	9.1(8.2-10.0)	5.8 (5.1-6.5)	4.6 (4.0-5.2)	5.1(4.4-5.8)	8.1(8.1-9.9)
Age-standadized^a^	4006	8.7 (7.8-9.6)	5.4 (4.7-6.1)	4.0 (3.4-4.6)	5.1 (4.4-5.8)	8.1 (7.3-8.9)
**Women**						
20-29	1171	3.4	2.7	0.9	0.4	0.7
30-39	1464	5.4	6.9	2.6	2.1	3.0
40-49	1131	7.8	9.7	6.0	6.9	8.7
50-59	926	9.4	9.9	7.3	8.4	17.5
60-69	664	9.1	9.6	7.6	9.8	25.4
≥70	127	4.9	15.5	7.4	6.1	27.2
Un-standardized	5483	6.6 (5.9-7.3)	7.6 (6.9-8.3)	4.6 (4.0-5.2)	49 (4.3-5.5)	9.4 (8.6-10.2)
Age-standardized^a^	5483	6.3 (5.7-6.9)	76 (6.9-8.3)	4.5 (4.0-5.0)	4.7 (4.1-5.3)	10 (9.2-10.8)
**Total (95%CI)**						
Un-standardized	9489	7.7 (7.2-8.2)	6.8 (6.3-7.3)	4.6 (4.2-5.0)	5.0 (4.6-5.4)	9.2 (8.6-9.8)
Age-standardized^a^	9489	7.3 (6.8-7.8)	6.7 (6.2-7.2)	4.2 (3.8-4.6)	4.9 (4.5-5.3)	9.1 (8.5-9.7)

### BMI, WHR and WHtR tertiles and prevalence of diabetes and prediabetes

Based on the sex-specific BMI, WHR and WHtR tertiles, a basically consistent trend was found of increasing prevalence of both diabetes and prediabetes with increasing BMI, WHR and WHtR tertiles (Table 4). Prevalence of diabetes and prediabetes increased with BMI tertiles, but no significant differences were found among BMI tertiles in men (Table 4).

**Table 4:** Distribution of diabetes and prediabetes across sex groups and BMI, WHR and WHtR tertiles

	**Man**			**Women**			**Total**		
**Age**	**Diabetes**	**Prediabetes**	**Estimated population (n=86161)**	**Diabetes**	**Prediabetes**	**Estimated population**	**Diabetes**	**Prediabetes**	**Estimated population (n=172280)**
30~	3.4	6.9	25641	7.1	3.6	25268	5.3	5.3	50909
40~	5.8	13.3	25571	10.8	7.6	25594	8.7	10.1	51165
50~	17.2	9.0	15662	10.8	18.0	15790	13.5	14.2	31452
60~	8.1	22.5	9759	18.4	17.5	9174	13.1	20.1	18933
70~	10.3	10.3	6279	20.9	23.3	6295	14.9	15.8	12574
80~	21.1	15.8	3249	23.5	35.5	3998	22.2	25.0	7247
Crude (95% CI)	10.0(7.5 -12.5)	13.3(10.4 -16.2)		13.1(10.4-14.0)	14.4(11.4-17.3)		11.6(9.8-13.4)	13.9(11.9-15.9)	
Age-standardized (95% CI) ^a^	8.3(8.1-9.5)	11.5(11.3-11.7)		11.9(11.7-12.1)	11.8(11.6-12.0)		10.0(9.8-10.1)	11.6(11.5-11.7	

P^*^ for Pearson Chi-Square test.

P^**^ for linear-by-linear association.

*WHR*, Waist hip ratio.

*WHtR*, Waist-to-height ratio.

*BMI*, body mass index.

*1*^*st*^: first.

*2*^*nd*^: second.

*3*^*rd*^: third.

### Associations between BMI, WHR and WHtR and diabetes

**Figure 1:**
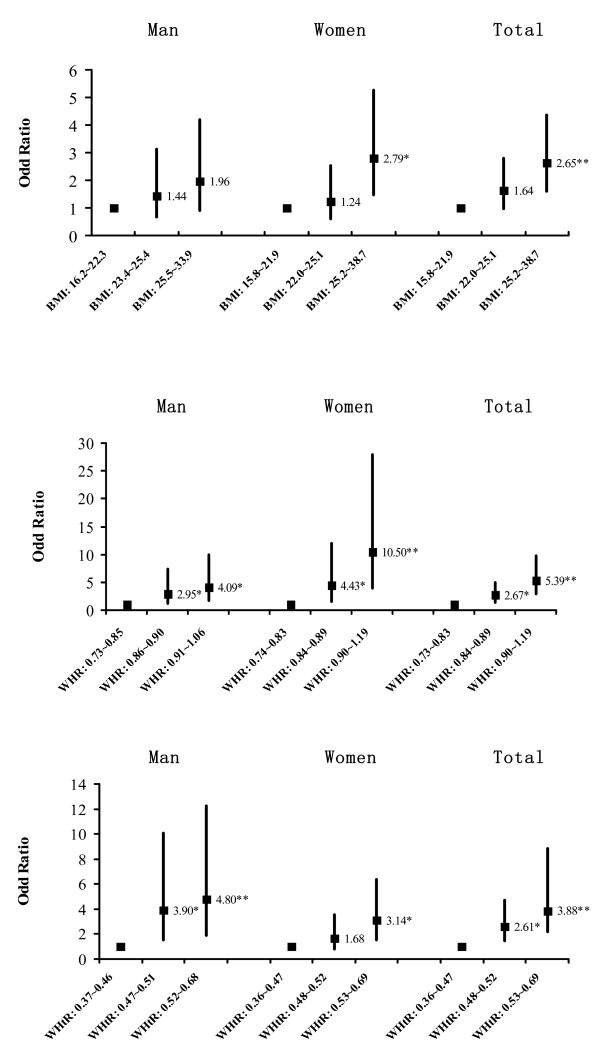
**Age adjusted odd ratios and 95% confidence intervals for diabetes by sex-specific BMI, WHR and WHtR tertiles. **(* P < 0.05, ** P < 0.001).

Logistic regression analyses were carried out separately in men and women to investigate the independent relation between anthropometric indices and diabetes after adjusting for age. Relative to 1^st^ BMI tertile, adjusted odds of diabetes significantly rose in 3^rd^ BMI tertile in men and total participants (Figure 1a). There was a consistent trend of increasing WHR and WHtR tertiles with increasing risk of diabetes. Compared to 1^st^ WHR tertile in women, there was a nearly tenfold increase in the risk of diabetes with 3^rd^ WHR tertile (OR 10.50, 95% CI 3.95-27.86) (Figure 1b). Men with 3^rd^ BMI tertile had 4.8-fold risk of getting diabetes compared to men with 1^st^ WHtR tertile (OR 4.798, 95% CI 1.88 ~ 12.26) (Figure 1c).

**Figure 2:**
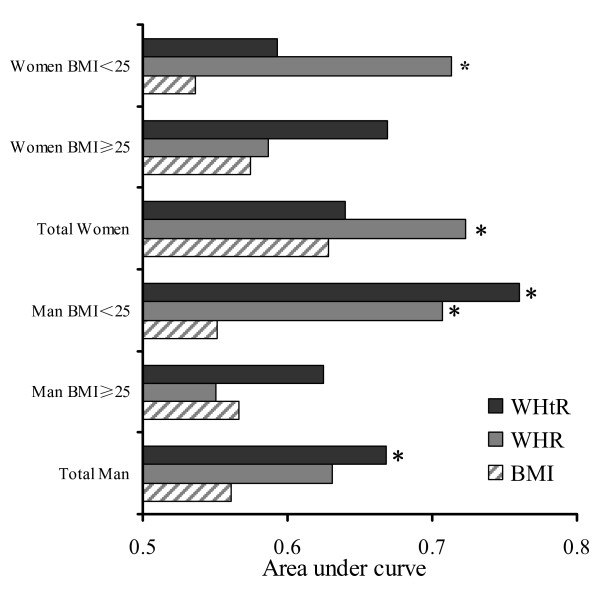
**Area under curve for anthropometric variables in predicting diabetes in man, women and subjects stratified by BMI. **(* P < 0.05 compared with BMI). *BMI*, body mass index. *WHR*, waist hip ratio. *WHtR*, waist:height ratio.

Figure 2 showed that only WHtR had significantly higher ROC area than BMI in total men (0.668 vs. 0.561, p < 0.05). In total women, however, only WHR had significantly higher ROC area than BMI (0.723 vs. 0.628, p < 0.05). BMI-stratified analyses (BMI < 25 kg/m^2^ and BMI ≥ 25 kg/m^2^) were performed. In both men and women with BMI ≥ 25 kg/m^2^, no significant difference was shown in the AUC among WHR, BMI and WHtR. In men with BMI < 25 kg/m^2^, both WHtR (0.760 vs. 0.551, P < 0.05) and WHR (0.707 vs. 0.551, P < 0.05) had significantly higher AUC than BMI (Figure 2). In women with BMI < 25 kg/m^2^, WHR had significantly higher AUC than BMI (0.713 vs. 0.536, P < 0.05)

Partial correlation analyses controlling for waist circumference revealed that BMI was not found to have any significant association with fasting plasma glucose and 2-h plasma glucose in both men and women (Table 5). In women, only WHR had significant correlation with fasting plasma glucose (r = 0.132, p < 0.000) and 2-h plasma glucose (r =0.162, p < 0.000). While controlling for waist circumference, WHtR had a much stronger association with both fasting plasma glucose and 2-h plasma glucose than WHR in men (Table 5).

**Table 5:** Waist circumference adjusted partial correlation coefficients between anthropometric variable and plasma glucose

	**Man**				**Women**		**Total**			
	**BMI**	**WHtR**	**WHR**	**BMI**	**WHtR**	**WHR**	**BMI**	**WHtR**	**WHR**	
Fasting plasma glucose	r=0.044 (P=0.332)	r=0.305 (P=0.000)	r=0.103 (P=0.024)	r=0.041 (P=0.336)	r=0.031 (P=0.473)	r=0.132 (P=0.002)	r=0.015 (P=0.622)	r=0.153 (P=0.000)	r=0.151 (P=0.000)	
2-h plasma glucose	r=0.017 (P=0.701)	r=0.303 (P=0.000)	r =0.148 (P=0.001)	r=0.044 (P=0.332)	r =0.036 (P=0.440)	r =0.162 (P=0.000)	r =0.014 (P=0.653)	r =0.200 (P=0.000)	r =0.116 (P=0.000)	

*BMI*: body mass index.

*WHR*: Waist hip ratio.

*WHtR*: Waist-to-height ratio.

## Discussion

### High prevalence of diabetes and prediabetes

In this study, we found that the prevalences of diabetes and prediabetes were 10.0% and 11.6% respectively. Yang and colleagues [3] reported that the total prevalence of diabetes and prediabetes in China had now reached 9.7% and 15.5% among adults. In the underdeveloped rural regions of China, the prevalence of diabetes and prediabetes were 5.8% and 15.9% [3]. These findings indicated that diabetes was very common in China, especially in underdeveloped rural areas. In addition,prevalence of diabetes and impaired glucose tolerance in all Asian countries were also high [14,15] and were expected to increase further during the next two decades. Yunnan is adjacent to SEAR and, therefore, we speculated that population in this region might share common risk factors for diabetes, such as common genetic determinants [16], food habit [17-19], climate and environment [19].

Several reasons may contribute to high prevalence of diabetes in these areas. Firstly, Asian populations, especially those of SEAR descent, are more prone to abdominal obesity and low muscle mass with increased insulin resistance [20]. Secondly, rapid socioeconomic development has led to a concurrent shift in infrastructure, technology, and food supply that promote overnutrition and sedentary lifestyles [2]. Polished rice and refined wheat, for example, form the basis of most Asian diets with high glycemic index and glycemic load values [20]. Increased urbanization and use of automobiles have caused many inhabitants to shift from a physically active, agrarian lifestyle marked by energy scarcity to a sedentary lifestyle. Thirdly, Songming inhabitants like cigarette smoking as well as salty and greasy food Smoking was known to induce insulin resistance and inadequate compensatory insulin secretion responses. A mete-analysis found that current smoking was associated with 44% increased risk of developing diabetes [21]. And some studies also found a positive association between smoking and diabetes [22-24].

### Undiagnosed diabetes in southwest rural areas of China

There was a lack of diabetes awareness in rural areas of China, which led to high prevalence of undiagnosed diabetes. Several studies have shown that the prevalence of undiagnosed diabetes was equal to or higher than diagnosed diabetes [25-27]. Our results were similar to results among Australians (47%) [28] and Chinese (46.6%) [3].

In rural areas of China, limited infrastructure for the care of diabetes and low diabetes awareness among the general public exacerbated the high prevalence of undiagnosed diabetes. In recent years, people in these areas have experienced reduced access to medical care. In fact, the number of village health officials has been reduced and the number of health-care centres has also decreased significantly in townships and villages [2]. Songming is situated in a mountainous area and is relatively undeveloped Consequently, it is difficult for local residents to have proper access to healthcare facilities or medicine.

#### Associations between BMI, WHR and WHtR and risk for diabetes

Overweight and obesity have been major risk factors for diabetes. The World Health Organization suggested the cutoff value of obesity as BMI ≥ 25 kg/m^2^ in Asia-Pacific region [29]**.** In the literature, a variety of anthropometric indicators have been suggested to predict diabetes and no consensus has been reached [30,31]. The reason may be the fact that predictive power of each anthropometric index was population-dependent [32] and varied with different ethnic groups [33].

The findings of this study showed WHtR and WHR were more efficient than BMI in identifying risk of diabetes. BMI had advantages and disadvantages in identifying overweight and obesity. Although BMI is by far the most widely used measurement to reflect general obesity and easy to measure, it does not accurately apply to elderly populations, pregnant women or very muscular athletes such as weight lifters. WHR and WHtR were used as indices of central obesity or abdominal obesity.

Abdominal obesity is closely correlated with both insulin resistance and diabetes. Abdominal fat is especially active hormonally, secreting adipokines that may possibly impair glucose tolerance [34], such as tumor necrosis factor α, interleukin-6, or resistin [35]. Those cytokines and low grade chronic inflammation in fat led to insulin resistance and pancreatic β cell damage and aggravated the development and progression of diabetes. Abdominal obesity is a characteristic feature in many Asian populations, especially in southeast Asia. So indices of central obesity such as WHR and WHtR are better than indices of general obesity such as BMI in identifying risk of diabetes.

Another interesting finding of this study was that the association of central obesity indices and risk of diabetes varied with gender. The strongest predictor of diabetes was WHR for the female subgroup and WHtR for the male subgroup.

This result in men was similar to previous studies. consistent with a study from Iran, WHtR appeared to be a better diabetes predictor in a follow-up of adult men from Iran [36]. Besides, a meta-analysis [37] containing 25 studies from nine countries (six Asian countries, the other three were in Caribbean, Europe, and Middle East) analyzing the data of AUCs in predicting type 2 diabetes showed significant differences between WHtR and BMI in only men (0. 672 vs. 0.726, P < 0.01). And our findings were consistent with previous studies in women. In Shanghai Women’s Health Study [38], risk of type 2 diabetes was more strongly related to WHR than with BMI. In Iowa Women’s Study [39], the dose–response relationship between risk of diabetes and WHR was much stronger than that with BMI.

WHR and WHtR are common indices of central obesity. Central obesity is closely correlated with both insulin resistance and diabetes. It is an excess accumulation of fat in the abdominal area. Obese men tend to be “apple-shaped” with waist circumference increasing. But obese women tend to be “pear-shaped”. Female had big pelvis designed for childbirth. Estrogens affect body fat distribution [40] causing fat to be stored in the buttocks in women. In the same WHtR values, an increasing WHR was more correlated to the increasing abdominal fat accumulation in women than in men and increased the risk of insulin resistance and diabetes. Furthermore, in men, we found that venous blood glucose and WHtR had a correlation stronger than that with WHR when waist circumference was adjusted. But in women, only WHR had significant association with venous blood glucose when waist circumference was adjusted for (Table 5). These data were in accordance with what we had speculated.

### Strengths and limitations

The strengths of this study are undiagnosed diabetes was established on OGTT, and a multistage stratified sampling design could select representative samples. To eliminate variability of OGTT, participants were told to undergo another OGTT in the hospital if undiagnosed diabetes were suspected. There were some limitations to this study. First, young people were undersampled and the mean age of all participants at the time of the study was 53.9 years. This may be due to the fact that many young people had gone to unversity or were working in cities. Second, the occupation and physical activity information were not collected in this study.

## Conclusions

With rapid economic growth in China, high prevalence of diabetes and prediabetes were found in this underdeveloped region. About half of the total cases with diabetes were undiagnosed. The association of obesity indices and diabetic risk factors varied with gender. The strongest predictor of diabetes was WHR for the female subgroup and WHtR for the male subgroup.

## Abbreviations

OGTT: Oral glucose tolerance test; BMI: Body mass index; WHR: Waist hip ratio; WHtR: Waist-to-height ratio; ROC: Receiver operating characteristic; CI: Confidence interval; IGT: Impaired glucose tolerance; IFG: Impaired fasting glucose.

## Competing interests

The authors state that they have no conflict of interest.

## Authors’ contributions

All authors participated in the design of the study. Author 1 were responsible for study design and protocol development. XL Zhao and XM Zhu analyzed the data and all authors interpreted findings and wrote the paper. Author 2 were involved in the preparation of instrument and laboratory work. Author 3 were involved in the study management. All authors have read this manuscript and approved the final version.

## Authors’ information

Xiaolong Zhao, Xiaoming Zhu, Weiwei Zhao, and Yiming Li: Department of Endocrinology & Metabolism, Huashan Hospital, Shanghai Medical College of Fudan University, Shanghai, China. Han-shen Zhang, Jinghui Li, Yonghui Shu, Songwu Li, and Minghui Yang: Internal Medicine Department, People’s Hospital, Songming County, Yunnan Province, China. Linghu Cai and Jiping Zhou: Center for disease control and prevention of Songming country, Yunnan province, China. Xiaolong Zhao and Xiaoming Zhu are co-first author.

## Pre-publication history

The pre-publication history for this paper can be accessed here:

http://www.biomedcentral.com/1471-2458/12/821/prepub

## References

[B1] Wild S Roglic G Green A Sicree R King H Global prevalence of diabetes: estimates for the year 2000 and projections for 2030 Diabetes Care 2004 27 1047 1053 10.2337/diacare.27.5.1047 15111519

[B2] Pan C Diabetes care in China: meeting the challenge World Hosp Health Serv 2005 41 29 30 32. 16300163

[B3] Yang W Lu J Weng J Jia W Ji L Xiao J Shan Z Liu J Tian H Ji Q Zhu D Ge J Lin L Chen L Guo X Zhao Z Li Q Zhou Z Shan G He J China National Diabetes and Metabolic Disorders Study Group, Prevalence of diabetes among men and women in China N Engl J Med 2010 362 1090 1101 10.1056/NEJMoa0908292 20335585

[B4] Li LM Rao KQ Kong LZ Yao CH Xiang HD Zhai FY Ma GS Yang XG Technical Working Group of China National Nutrition and Health Survey. A description on the Chinese national nutrition and health survey in 2002 Zhonghua Liu Xing Bing Xue Za Zhi 2005 26 478 484 16334996

[B5] Bin yang Between Winds and Clouds: The Making of Yunnan (Second Century BCE-Twentieth Century CE) 2009 New York: Columbia University Press

[B6] Zhou H Fiscal decentralization and the development of the tobacco industry in China China Econ Rev 2000 11 114 133 10.1016/S1043-951X(00)00013-4

[B7] Xu H Song Y You NC Zhang ZF Greenland S Ford ES He L Liu S Prevalence and clustering of metabolic risk factors for type 2 diabetes among Chinese adults in Shanghai China. BMC Public Health 2010 10 683 10.1186/1471-2458-10-683 PMC2989965 21062480

[B8] National islet β-cell study group of China Plasma glucose, insulin and C-peptide changes in normal subjects during steamed-bread meal test Zhonghua Yi Xue Za Zhi 1982 62 643 647 6819873

[B9] Wolever TM Chiasson JL Csima A Hunt JA Palmason C Ross SA Ryan EA Variation of postprandial plasma glucose, palatability, and symptoms associated with a standardized mixed test meal versus 75 g oral glucose Diabetes Care 1998 21 336 340 10.2337/diacare.21.3.336 9540012

[B10] Perloff D Grim C Flack J Frohlich ED Hill M McDonald M Morgenstern BZ Human blood pressure determination by sphygmomanometry Circulation 1993 88 2460 2470 10.1161/01.CIR.88.5.2460 8222141

[B11] Liu Y Tong G Tong W Lu L Qin X Can body mass index, waist circumference, waist-hip ratio and waist-height ratio predict the presence of multiple metabolic risk factors in Chinese subjects? BMC Public Health 2011 11 35 10.1186/1471-2458-11-35 21226967 PMC3032682

[B12] Department of Noncommunicable Disease Surveillance Definition, diagnosis and classification of diabetes mellitus and its complications: report of a WHO consultation. Part 1. Diagnosis and classification of diabetesmellitus 1999 Geneva: World Health Organization

[B13] Hennekens CH Buring JE Mayrent SL Epidemiology in medicine 1987 Boston: Little Brown and Company 90 93

[B14] Letchuman GR Wan Nazaimoon WM Wan Mohamad WB Chandran LR Tee GH Jamaiyah H Isa MR Zanariah H Fatanah I Ahmad Faudzi Y Prevalence of diabetes in the Malaysian National Health Morbidity Survey III 2006 Med J Malaysia 2010 65 180 186 21939164

[B15] Aekplakorn W Abbott-Klafter J Premgamone A Dhanamun B Chaikittiporn C Chongsuvivatwong V Suwanprapisa T Chaipornsupaisan W Tiptaradol S Lim SS Prevalence and management of diabetes and associated risk factors by regions of Thailand: Third National Health Examination Survey 2004 Diabetes Care 2007 30 2007 2012 10.2337/dc06-2319 17468342

[B16] Jin L Su B Natives or immigrants: modern human origin in east Asia Nat Rev Genet 2000 1 126 133 10.1038/35038565 11253652

[B17] Janowski M Kerlogue F Kinship and Food in South East Asia 2007 NIAS Press

[B18] Zhao R Yunnan’s Dietary Culture in the great space-time view Dietetic Culture Research 2007 23 13 20

[B19] Hoanh CT Guttman H Droogers P Aerts J *Water, climate, food, and environment in the Mekong Basin in Southeast Asia* ADAPT Final Report 2003 International Water Management Institute, Mekong River Commission Secretariat and Institute of Environmental Studies

[B20] Ludwig DS The glycemic index: physiological mechanisms relating to obesity, diabetes, and cardiovascular disease JAMA 2002 287 2414 2423 10.1001/jama.287.18.2414 11988062

[B21] Willi C Bodenmann P Ghali WA Faris PD Cornuz J Active smoking and the risk of type 2 diabetes: a systematic review and meta-analysis JAMA 2007 298 2654 2664 10.1001/jama.298.22.2654 18073361

[B22] Hur NW Kim HC Nam CM Jee SH Lee HC Suh I Smoking cessation and risk of type 2 diabetes mellitus: Korea Medical Insurance Corporation Study Eur J Cardiovasc Prev Rehabil 2007 14 244 249 10.1097/01.hjr.0000239474.41379.79 17446803

[B23] Chen CC Li TC Chang PC Liu CS Lin WY Wu MT Li CI Lai MM Lin CC Association among cigarette smoking, metabolic syndrome, and its individual components: the metabolic syndrome study in Taiwan Metabolism 2008 57 544 548 10.1016/j.metabol.2007.11.018 18328358

[B24] Ko GT Chan JC Tsang LW Critchley JA Cockram CS Smoking and diabetes in Chinese men Postgrad Med J 2001 77 240 243 10.1136/pmj.77.906.240 11264486 PMC1742000

[B25] Mooy JM Grootenhuis PA de Vries H Valkenburg HA Bouter LM Kostense PJ Heine RJ Prevalence and determinants of glucose intolerance in a Dutch Caucasian population The Hoorn Study. Diabetes Care 1995 18 1270 1273 10.2337/diacare.18.9.1270 8612442

[B26] Will new diagnostic criteria for diabetes mellitus change phenotype of patients with diabetes? Reanalysis of European epidemiological data. DECODE Study Group on behalf of the European Diabetes Epidemiology Study Group. BMJ 1998 317 371 375 10.1136/bmj.317.7155.371 9694750 PMC28629

[B27] Mohan V Deepa M Deepa R Shanthirani CS Farooq S Ganesan A Datta M Secular trends in the prevalence of diabetes and impaired glucose tolerance in urban South India--the Chennai Urban Rural Epidemiology Study (CURES-17) Diabetologia 2006 **49.** 10.1007/s00125-006-0219-2 16570158

[B28] Dunstan DW Zimmet PZ Welborn TA De Courten MP Cameron AJ Sicree RA Dwyer T Colagiuri S Jolley D Knuiman M Atkins R Shaw JE The rising prevalence of diabetes and impaired glucose tolerance: the Australian Diabetes, Obesity and Lifestyle Study Diabetes Care 2002 25 829 834 10.2337/diacare.25.5.829 11978676

[B29] World Health Organization The Asia-Pacific perspective: redefining obesity and its treatment February 2000 http://www.wpro.who.int/nutrition/documents/Redefining_obesity/en/index.html.

[B30] Taylor AE Ebrahim S Ben-Shlomo Y Martin RM Whincup PH Yarnell JW Wannamethee SG Lawlor DA Comparison of the associations of body mass index and measures of central adiposity and fat mass with coronary heart disease, diabetes, and all-cause mortality: a study using data from 4 UK cohorts Am J Clin Nutr 2010 91 547 556 10.3945/ajcn.2009.28757 20089729

[B31] Qiao Q Nyamdorj R Is the association of type II diabetes with waist circumference or waist-to-hip ratio stronger than that with body mass index? Eur J Clin Nutr 2010 64 30 34 10.1038/ejcn.2009.93 19724291

[B32] Molarius A Seidell JC Selection of anthropometric indicators for classification of abdominal fatness – a critical review Int J Obes Relat Metab Disord 1998 22 719 727 10.1038/sj.ijo.0800660 9725630

[B33] Lear SA Chen MM Frohlich JJ Birmingham C The relationship between waist circumference and metabolic risk factors: cohorts of European and Chinese descent Metabolism 2002 51 1427 1432 10.1053/meta.2002.34042 12404193

[B34] Kern PA Ranganathan S Li C Wood L Ranganathan G Adipose tissue tumor necrosis factor and interleukin-6 expression in human obesity and insulin resistance Am J Physiol Endocrinol Metab 2001 280 E745 E751 11287357 10.1152/ajpendo.2001.280.5.E745

[B35] Hotamisligil GS Arner P Caro JF Atkinson RL Spiegelman BM Increasadipose tissue expression of tumor necrosis factor-alpha in human obesity and insulin resistance J Clin Invest 1995 95 2409 2415 10.1172/JCI117936 7738205 PMC295872

[B36] Hadaegh F Zabetian A Harati H Azizi F Waist/height ratio as a better predictor of type 2 diabetes compared to body mass index in Tehranian adult men–a 3.6-year prospective study Exp Clin Endocrinol Diabetes 2006 114 310 315 10.1055/s-2006-924123 16868890

[B37] Lee CM Huxley RR Wildiabetesan RP Woodward M Indices of abdominal obesity are better discriminators of cardiovascular risk factors than BMI: a meta-analysis J Clin Epidemiol 2008 61 646 653 10.1016/j.jclinepi.2007.08.012 18359190

[B38] Rosenthal AD Jin F Shu XO Yang G Elasy TA Chow WH Ji BT Xu HX Li Q Gao YT Zheng W Body fat distribution and risk of diabetes among Chinese women Int J Obes Relat Metab Disord 2004 28 594 599 10.1038/sj.ijo.0802597 14770196

[B39] Kaye SA Folsom AR Sprafka JM Prineas RJ Wallace RB Increased incidence of diabetes mellitus in relation to abdominal adiposity in older women J Clin Epidemiol 1991 44 329 334 10.1016/0895-4356(91)90044-A 1999691

[B40] Sites CK L’Hommedieu GD Toth MJ Brochu M Cooper BC Fairhurst PA The effect of hormone replacement therapy on body composition, body fat distribution, and insulin sensitivity in enopausal women: a randomized, double-blind, placebo-controlled trial J Clin Endocrinol Metab 2005 90 2701 2707 10.1210/jc.2004-1479 15687338

[B41] World Health Organization (WHO) Waist circumference and waist-hip ratio: report of a WHO expert consultation. Geneva, 8–11 December 2008 2011 Geneva: WHO

[B42] He Y Zhai F Ma G Feskens EJ Zhang J Fu P Van’t Veer P Yang X Abdominal obesity and the prevalence of diabetes and intermediate hyperglycaemia in Chinese adults Public Health Nutr 2009 12 1078 1084 10.1017/S1368980008003856 18986591

